# Endodontic Treatment of a Mandibular Second Molar with Two Mesial Roots: Report of a Case

**Published:** 2008-10-01

**Authors:** Shohreh Ravanshad, Mohammad Reza Nabavizade

**Affiliations:** 1 *Department of Endodontics, Dental School, Shiraz University of Medical Sciences, Shiraz, Iran*

**Keywords:** Anatomy, Mandibular, Root, Second Molar

## Abstract

A case of unusual root morphology is presented to demonstrate anatomic variations in mandibular second molars. The most common configuration of mandibular second molar is to have two roots with three root canals; however mandibular molars may have many different combinations. Endodontic therapy was performed in a mandibular second molar with 3 separate roots 2 located mesially and one distally. Radiographically all 3 root canals terminated with individual foramina. Three orifices or 3 independent canals were found in the 3 separate roots, indicating a rare anatomic configuration. Looking for additional roots, canals and unusual morphology is an important part of successful endodontics as the knowledge of their existence occasionally enable clinicians to treat a case that otherwise might have ended in failure.

## INTRODUCTION

One of the most important aspects in current endodontics is a thorough knowledge of internal root anatomy. This aspect, together with a correct diagnosis and appropriate cleaning and shaping of the root canal system, will usually lead to a successful outcome. Slowey emphasized that root canal morphology was limitless in its variability and that clinicians must be aware that anatomic variations constitute an impressive challenge to endodontic success. Undetected extra roots or root canals are a major reason for failure of root canal treatment (1).

A clear understanding of root morphology and canal anatomy is an essential prerequisite to achieving clean, disinfected and 3-dimensionally obturated root canal systems (2). Many of the challenges faced during root canal treatment may be directly attributed to an inadequate understanding of the tooth morphology. Human molars show considerable anatomic variation and abnormalities with respect to number of roots and root canals. Unusual tooth anatomy associated with the mandibular molars has been investigated in several studies (2-7).

Manning has studied the root canal anatomy of 149 extracted mandibular second molars using clearing technique. He found that 22%had single roots, 76% had two roots, and 2% had three roots (8). Costa Rocha *et al.* studied the external and internal anatomy of 628 extracted mandibular first and second molars. Analysis of mandibular second molars root showed that 84.1% presented two separate roots, 15.9% fused roots and 1.5% three roots (9). The anatomy of mandibular second molar has racial variations; using periapical radiographs of 328 patients (105, Mongoloid origin; 106, Negro; 117, Caucasian), Ferraz and Pécora reported an incidence of three-rooted mandibular second molar in 2.8% of patients of Mongoloid origin, 1.8% of Negro origin, and 1.7% Caucasian (10). Radiographs are an important and necessary aid in RCT, and accurate radiographic techniques and proper interpretation are essential for sound diagnosis and treatment. The use of preoperative radiographs is the best way to detect and evaluate root canal morphology and anatomy. Further radiographs should be taken at different angles to confirm any variation in anatomical features (11).

**Figure 1:**
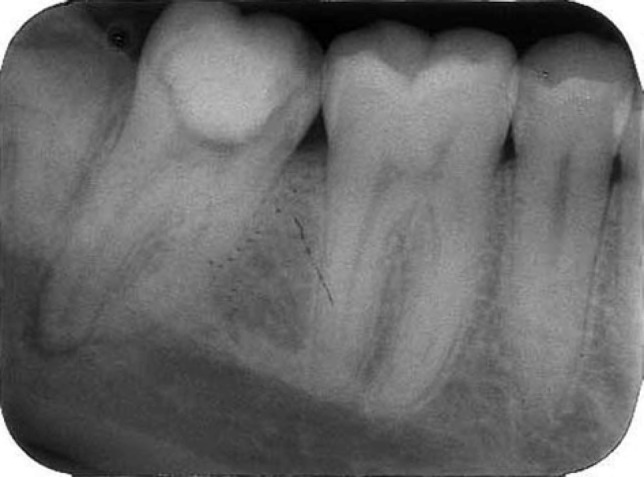
A pulpotomized right mandibular second molar. Careful examination of the radiographs revealed the possibility of more than two roots

With increasing reports of aberrant canal morphology, the clinician needs to be aware of this variability. The purpose of this clinical report is to show root canal treatment of a mandibular second molar with two mesial roots detected during routine root canal treatment.

## CASE REPORT

A 27-year-old male patient was referred with constant pain of the right mandibular second molar for root canal treatment to the postgraduate Endodontic Department of Shiraz Dental School; Shiraz university of Medical Sciences. A general dental practitioner had started root canal treatment. The tooth was symptomatic, with the patient complaining of severe pain. A diagnostic radiograph revealed a pulpotomized tooth with a temporary filling. Periodontal pockets were within normal limit. The medical history was noncontributory. Careful examination of the radiographs revealed the possibility of more than two roots (Figure 1). 

**Figure 2:**
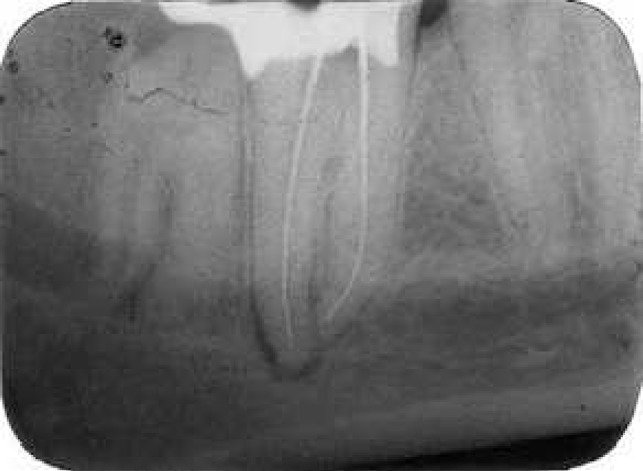
The working length measurement radiograph showed three files in three separated roots

The tooth was anesthetized and isolated with a rubber dam; the temporary filling was removed using a round diamond bur, to gain good access to the pulp chamber. Clinical evaluation of the internal anatomy confirmed the presence of three root canal orifices, two located mesially and one distally. On closer inspection with ×4.5 magnification prismatic loupes (Zeiss Eyemag Pro S; Carl Zeiss SpA, Arese, Italy) the pulp chamber floor showed 3 orifices corresponding to 3 root canals. The working lengths of each canal were estimated by means of Root ZX electronic apex locator (J. Morita Corporation, Tokyo, Japan), then confirmed by a radiograph (distal canal=31 mm, mesial canals =29 and 27 mm). The working length measurement radiograph showed three independent root canals in three separated roots (Figure 2).

The canals were initially instrumented with #15 nickel-titanium files (Dentsply Maillefer, Ballaigues, Switzerland) under irrigation with 2.5% sodium hypochlorite (NaOCl), coronal flaring was carried out using # 2 and 3 Gates Glidden burs (Dentsply, Maillefer, Ballaigues, Switzerland). All canals were cleaned and prepared by hand with nickel-titanium files using a crown-down technique similar to that described by Saunders and Saunders (12).

**Figure 3:**
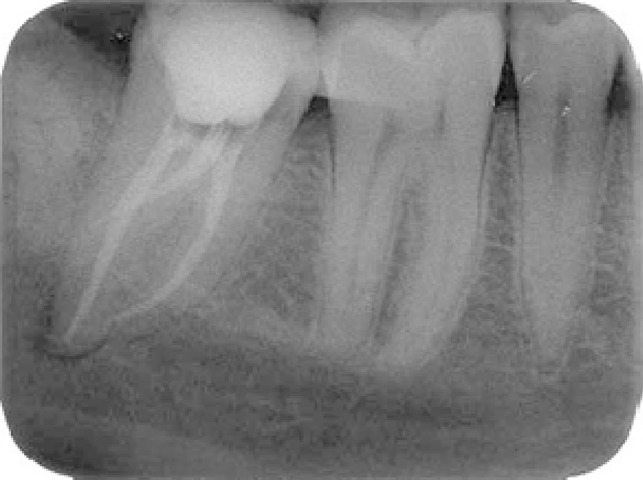
Postoperative radiographs. Three separated root canals were filled with gutta-percha and sealer

Root canal treatment was scheduled over two visits because of the complexity of the root canal systems. Calcium hydroxide paste was used as an intracanal medicament. A sterile pellet was placed in the pulp chamber, and Coltosol (Coltene, Altstatten, Switzerland) was placed over the access cavity as a temporary filling to prevent coronal leakage. One week later, at the second appointment, all symptoms had disappeared. All three canals were obturated with Tubli-seal (Kerr UK, Peterborough, UK) and laterally condensed gutta-percha points. Final radiographs were taken to establish the quality of the obturation (Figure 3). After completion of root canal treatment, the tooth filled with temporary cement and referred to restorative department for final restoration.

## DISCUSSION

The development of various areas of dentistry requires precise study of morphology of human teeth so that better oral health can be provided. The mandibular molars play a principal role in mastication and help to maintain the vertical dimension of the face, continuity of the dental arch, maintaining the cheeks and tongue in their position and therefore maintaining the stomatognathic function.

Studies of the internal and external anatomy of teeth have shown that anatomical variations can occur in all group types, in individuals and in various racial groups. Anatomical variations should be expected as a frequent possibility. The clinician must therefore be fully aware of dental morphology in order to provide better care (9).

Most endodontic text books and dental anatomy books, describe the mandibular second molar as having two roots one mesial and one distal with two, three or four root canals (13-16). However, a mandibular second molar with a conical root and wide single root canal is also reported (17). Weine stated that this tooth may have more anatomical variations than all other molar teeth. When only one root is present, the root canal system may present only a broad root canal, two canals that may or may not join, or a C-shaped canal (18).

Researchers have shown that the anatomy of mandibular molars requires much attention since the number of roots and canals are quite variable. Maggiore *et al.* also noted that the roots of the mandibular second molar can vary from one to three roots (19). Manning reported three out of 149 mandibular second molars had three roots (8). Costa Rocha *et al.* in analysis of mandibular second molars root showed six teeth out of a total of 396 with three roots (9). The anatomy of human teeth present racial variations; according to Ferraz and Pécora's report, three-rooted mandibular second molars were found in 3 teeth of Mongoloid origin, 2 teeth of Negro origin, and 2 of Caucasian origin patients (10).

In certain circumstances, root canals may be left untreated during endodontic therapy if the practitioner is unable to detect their presence (20). The presence of extra roots is readily determined using routine radiography, as demonstrated in the current case. Knowledge of anatomic aberrations, such as root position, root shape and relative root outline will also help to decrease the failure rate of root canal therapy. From a clinical standpoint, when the initial radiograph shows the image of an unusual anatomic form it is recommended to take a second radiograph for additional information particularly with a mesial or distal projection (11).

The conventional root canal anatomy indicates the location of the initial access. The knowledge of both the normal and abnormal anatomy of molars shows the parameters under which root canal therapy is to be performed and can directly modify the probability of success. This is the reason why clinicians should be aware that variations in tooth morphology may well occur. Failure to treat a canal is an obvious reason for root canal treatment failure. Therefore, all clinicians must make every effort to locate and treat all existing canals during endodontic treatment. Our case report was a rare case that showed two mesial roots in a mandibular second molar. It elaborates the importance of adequate knowledge about root canal morphology for a proper complete endodontic treatment of such three root canalled teeth.

## CONCLUSION

Anatomic variation in the number of roots and root canals can occur in any tooth. Examination of clear radiographs taken from different angles and careful evaluation of the internal anatomy of teeth is essential. Root canal treatment is likely to fail if extra roots or root canals are not detected.
